# Transient Administration of Dopaminergic Precursor Causes Inheritable Overfeeding Behavior in Young *Drosophila melanogaster* Adults

**DOI:** 10.3390/brainsci10080487

**Published:** 2020-07-28

**Authors:** Thiago C. Moulin, Federico Ferro, Samuel Berkins, Angela Hoyer, Michael J. Williams, Helgi B. Schiöth

**Affiliations:** 1 Functional Pharmacology Unit, Department of Neuroscience, Uppsala University, 751 24 Uppsala, Sweden; FEDERICO.FERRO@studenti.units.it (F.F.); samuel.berkins@gmail.com (S.B.); angela.hoyer@gmx.de (A.H.); michael.williams@neuro.uu.se (M.J.W.); helgi.schioth@neuro.uu.se (H.B.S.); 2 Institute for Translational Medicine and Biotechnology, Sechenov First Moscow State Medical University, 119146 Moscow, Russia

**Keywords:** L-DOPA, dopamine, feeding, reward, energy intake, development

## Abstract

Imbalances in dopaminergic signaling during development have been indicated as part of the underlying neurobiology of several psychiatric illnesses, including schizophrenia, major depression, bipolar disorder, and food addiction. Yet, how transient manipulation of dopaminergic signaling influences long-lasting behavioral consequences, or if these modifications can induce inheritable traits, it is still not understood. In this study, we used the *Drosophila melanogaster* model to test if transient pharmacological activation of the dopaminergic system leads to modulations of feeding and locomotion in adult flies. We observed that transient administration of a dopaminergic precursor, levodopa, at 6 h, 3 days or 5 days post-eclosion, induced overfeeding behavior, while we did not find significant effects on locomotion. Moreover, this phenotype was inherited by the offspring of flies treated 6 h or 3 days post-eclosion, but not the offspring of those treated 5 days post-eclosion. These results indicate that transient alterations in dopaminergic signaling can produce behavioral alterations in adults, which can then be carried to descendants. These findings provide novel insights into the conditions in which environmental factors can produce transgenerational eating disorders.

## 1. Introduction

The dopaminergic system is involved in the pathophysiology of several psychiatric illnesses, such as schizophrenia [1], major depression [2], and bipolar disorder [3]. Transient disturbances in dopamine signaling during development have been implicated as a possible origin to maladaptive neural mechanisms, which in turn could generate aberrant behaviors [4]. Accordingly, activation of dopaminergic neurons during the developmental stages of *Drosophila melanogaster* models were shown to cause adult phenotypes related to cognitive impairments, such as alterations in sensory responsiveness and locomotion [5,6]. Mounting evidence also indicates that imbalances of the dopaminergic reward system facilitates the emergence of compulsive responses to food, contributing to food addiction and obesity through modulation of reinforcement and motivation [7,8,9,10]. However, the link between developmental dopaminergic disturbances and subsequent alterations in eating behavior remains surprisingly unexplored.

Moreover, it is suggested that changes in the parental environment during pregnancy can have significant phenotypic consequences on the next generation. One of the first documented cases of such transgenerational influence was the Dutch Hunger Winter Study, which showed that children conceived during a starvation period in the Netherlands had higher rates of obesity and diabetes [11,12]. Additionally, recent reports show that neurological and psychiatric conditions have a persistent impact on the following generations [13]. In *Drosophila*, exposure to factors such as stress or misbalanced nutrition in adults have also been shown to prompt a range of transgenerational behavioral effects, such as changes in ethanol preference [14], cold-temperature response [15], oviposition choice [16], and obesity-like phenotypes [17].

In the current study, we investigate if transient manipulation of dopaminergic pathways by levodopa treatment after eclosion is able to induce inheritable feeding-behavior alterations in the *Drosophila* melanogaster model. We examined if administering 1 mM of this dopaminergic precursor for 48 h in different early adult stages (6 h, 3 days, or 5 days post-eclosion) can induce later-life consequences (7 days post-eclosion). Then, we test if the first generation of the treated flies displayed similar phenotypes. Taken together, our results indicate that transitory dopaminergic imbalances can produce feeding disorders, which can be inherited by the offspring of parents affected during early adult stages.

## 2. Materials and Methods

### 2.1. Fly Strains and Maintenance

For the experiments, 6 h to 7 day-old wild-type CSORC-strain *Drosophila* melanogaster adults were used (originated from CantonS and OregonR-C flies, Bloomington Drosophila Stock Center, Bloomington, IN, USA) and kept at 25 °C, 12:12-hr light/dark, 60% humidity. The flies were fed by the Fisherbrand Jazz-Mix Drosophila food, complemented with 8.3% yeast extract (both from Fisher Scientific, Gothenburg, Sweden).

### 2.2. Pharmacological Treatment

**Figure 1:**
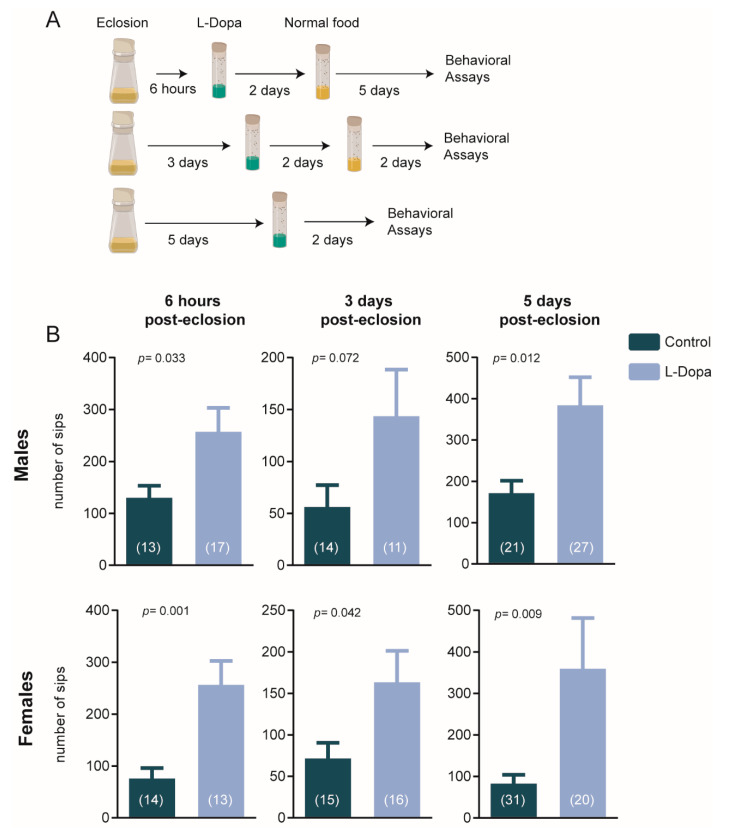
Dopaminergic administration induces overeating. (**A**) Before the behavioral testing at 7-days old, levodopa treatment was delivered in the food for 2 days to 6 h, 3 days or 5 days post-eclosion flies. (**B**) All groups showed increased feeding behavior after the transient dopaminergic manipulation, with only 3 days old -treated males slightly above statistical significance (Student *t*-test; alpha = 0.05). Bars and errors represent mean ± SEM, and the sample size for each group is given within brackets.

For age-controlled experimentation, CSORC cultures were cleared of adult flies, so only larvae and pupae remained, and then monitored for eclosion. At different time points post-eclosion (5 days, 3 days and, 6 h), male and female flies were collected and separately transferred to food containing 1 mM Levodopa (Sigma-Aldrich, Stockholm, Sweden) for 2 days. Levodopa was chosen as the treatment to induce alterations in dopaminergic signaling, as it is known to induce increases the levels of dopamine in the fly brain and to revert deficits caused by models of dopamine deficiency [18,19,20,21]. The intervention times were established based on previous reports, where it was shown that ~2.5 days of genetic-induced activation of dopaminergic neurons in pre-ecloded or young flies, but not adults, could produce future deficits in visual responsiveness [5,6]. Additionally, the levodopa concentration was chosen following in a previous study reporting that 48 h of administration of this drug could induce depression-like behavioral effects in adult *Drosophila* [22]. After treatment, the flies were transferred back to normal food until reaching 7 days-old, when they were tested (Figure 1A).

For transgenerational testing, the same treatment procedure was performed, but as the flies reached 7 days-old, males and females were placed together to mate for 3 days, and then removed from the vial. The feeding behavior and general activity of the offspring were then tested also at 7 days after eclosion.

### 2.3. Feeding Behavior

To assess feeding behavior, we used the FlyPad, a recently-developed equipment based on the proboscis interaction with the food [23]. Flies were put in an arena individually, with two different channels filled with 1–2 µL food, using capacitive sensors to detect sips taken by the fly. The control and treated groups were tested simultaneously under the same conditions. All feeding assays were executed for one to two hours, between 1 and 3 h after lights. For better detection of differences in feeding behavior, flies were starved for a period of 12–14 h previous to the experiment.

For the analyses, we chose the number of sips as the primary outcome, as this measure is shown to be strongly correlated with the ingested food volume [23]. For exploratory assessment secondary feeding behaviors, we extracted the number of activity bouts (i.e., how often an animal approaches the food) and the duration of the activity bouts (i.e., the average feeding duration for each approach), parameters also shown to correlate with food consumption.

### 2.4. General Activity and Sleep

For the general activity and sleep experiments, we used the Drosophila Activity Monitor System (DAMS, TriKinetics Inc., Waltham, MA, USA), as previously described [24,25]. Briefly, flies were anesthetized with CO2 and put individually in plastic tubes, which were then placed horizontally in the detection system. The tube ends were sealed with regular fly food at one end and a small cotton bud at the other end. Flies were maintained in a 12:12 light: dark cycle for 3 days, starting at lights on. Next, the raw data was converted to CSV files using the DamFileScan software. The Sleep and Circadian Analysis MATLAB Program (SCAMP) by Vecsey Lab was used to analyze the data and evaluate different aspects of activity behavior.

### 2.5. Statistical Analysis

To evaluate the effect of levodopa on males and female flies at different ages, we performed separate experiments comparing each treatment group with matched controls. As independent results, the group comparisons were analyzed using the two-sided Student’s *t*-test, considering alpha = 0.05. For transparency in the interpretation of these multiple comparisons, we report the exact *p*-values for all performed tests in the respective figures or tables. Likewise, the effect size, standard error, and sample sizes for each experiment are given in their respective figures or tables. Additionally, for the estimation of the overall effect of treatment, sex, and age, considering the interactions between these factors, we performed a least-squares multiple linear regression model and report the outcomes at Supplementary Table S1. All statistical analyses were performed using the GraphPad Prism 8 software.

## 3. Results

### 3.1. Transient Dopaminergic Disturbances Affect Feeding Behavior

We investigated whether a transient levodopa-increase in dopamine synthesis at different stages of *Drosophila* early adults could affect feeding behavior by assessing the number of fly interactions with the food (Figure 1A). Two days of pharmacological manipulation, at 6 h or 5 days post-eclosion, increased the number of sips when flies were tested at 7-days post-eclosion (Figure 1B). The overfeeding behavior observed was consistent and statistically significant between male and female flies, except for males 3 days post-eclosion, which might be explained by the reduced number of animals in this group. Linear regression analysis to estimate the overall effect of these factors indicates that levodopa treatment has a significant effect across groups (*p* = 0.0061), while age and sex had a non-significant influence on the model (Supplementary Table S1).

Additionally, exploratory analyses of secondary feeding behavior outcomes (Supplementary Table S2) show that both treated males and females display a higher number of interactions with the food (i.e., number of activity bouts, significant for all groups but 3 days post-eclosion treated flies), while only females have increased food ingestion within each interaction (i.e., activity bout duration, significant for all female flies).

### 3.2. Transient Dopaminergic Dysregulation Does Not Influence General Activity or Sleep

Next, we examined whether our pharmacological manipulation protocol was able to alter the locomotion or sleep in *Drosophila*. After monitoring three days of activity, starting 7 days post-eclosion, we could not find any differences in the average number of movements or sleep counts for the groups, independent of sex or developmental stage of treatment (Table 1).

### 3.3. Early Dopaminergic Application Affects Transgenerational Feeding Behavior 

**Figure 2:**
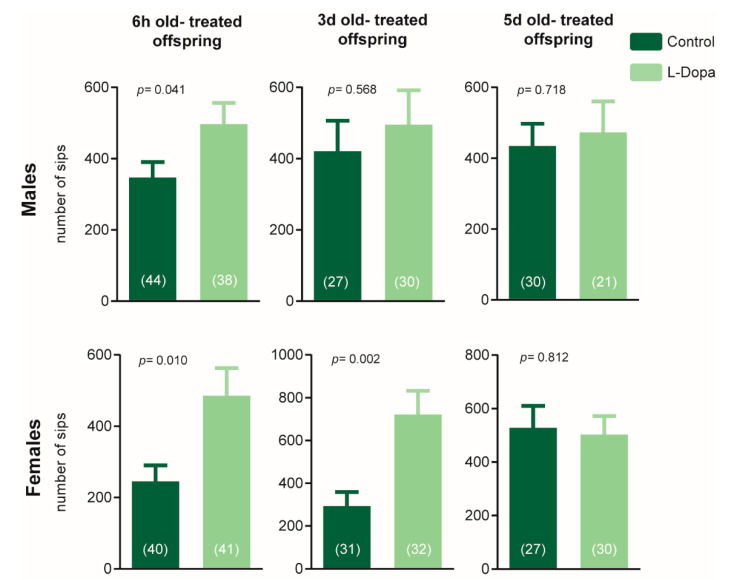
Parental dopaminergic administration 6 h and 3 days post-eclosion generates offspring overeating. The offspring of transiently-treated flies with levodopa after 6 h post-eclosion and the female progeny of 3 days old-treated flies display significantly increased feeding behavior (Student *t*-test; alpha = 0.05). No differences can be observed in the male descendants of 3 days old -treated flies and in the 5 days old -treated offspring. Bars and errors represent mean ± SEM, and the sample size for each group is given within brackets.

To access if the transient dopaminergic misbalance during different early adult stages could influence the offspring, we mated flies at 7 days post-eclosion, which had undergone levodopa administration, as shown in Figure 1A. The F1 generation was then tested at 7-days post-eclosion in the feeding behavior paradigm (Figure 2). We observed that both sexes of the F1 generation from flies treated at 6 h post-eclosion had similar overfeeding behavior, as they exhibited a significant increase in the number of sips. Moreover, there was a differential transgenerational influence of transient levodopa for males and females when it was given 3 days post-eclosion. Offspring female flies had a higher number of sips than controls, while male flies did not have a significant increase in feeding. Lastly, the F1 generation of flies treated with levodopa 5 days post-eclosion did not exhibit any significant phenotypes. Considering all results, the linear regression model indicates an overall effect of the treatment (*p* = 0.0318) on the number of sips, while age or sex factors were not statistically significant, likely due to the non-linear relations between age and sex.

The offspring did not show any particular behavioral pattern when analyzing the secondary assessment of activity bouts number and duration (Supplementary Table S3). Neither measure was altered for the offspring of flies treated 5 days post-eclosion. The number of activity bouts was significantly higher for the F1 generation females, but not males, of the 6 h-old and 3 days-old treated flies. In turn, activity bout duration was increased in 3 days post-eclosion F1 females and 6 h post-eclosion F1 males.

## 4. Discussion

In this study, we established a *Drosophila* melanogaster model of overfeeding by pharmacologically inducing imbalances in the dopaminergic system. The transient nature of this dopaminergic manipulation is intended to mimic temporary imbalances during development that may increase the likelihood of cognitive impairments later in life, including eating disorders [9]. We observed that transient dopaminergic manipulation via 48-h levodopa administration (1 mM) during different early adult stages could induce overfeeding behavior, indicated by a significant increase in the number of sips. This effect was consistent across male and female flies. However, complementary analysis of the feeding bout parameters indicate males and females might have different strategies for increasing food intake, as both sexes showed a higher number of interaction with the food, but only females spent more time within each interaction. Importantly, the overall activity and sleep/wake behavior assessment showed no differences between groups, indicating that the increase in food ingestion was not related to modifications in locomotion or disturbed sleep, which could by itself cause behavioral or metabolic consequences [26].

These results are consistent with the hypothesis that environmental factors during brain development could induce the erratic development of dopaminergic circuits, resulting in persistently altered dopamine signaling, which in turn could generate psychiatric disorders [1]. Notably, our observations differ from previous reports showing a reduction in food consumption immediately after levodopa administration in adult flies [22], indicating that compensatory mechanisms may be involved in the neuronal response to the 48-h application of this dopamine precursor. Accordingly, it is suggested that transient increase in dopaminergic activity during development prompts compensatory downregulation of dopamine receptors [6], implying a role for homeostatic regulation of the developing nervous system, thought to be necessary for correct brain circuitry formation [27,28]. In fact, synaptic changes in dopaminergic neurons due to disturbances in homeostatic processes are thought to be involved in the neurobiology of disorders like major depression and addiction [29,30]. However, the range of behavioral effects caused by misbalances in the developing dopamine system are still elusive.

Evidence shows that environmental disturbances can produce obesity-like transgenerational effects in both humans [11] and *Drosophila* [17]. We sought to evaluate if the offspring of levodopa-treated flies also had altered feeding behavior in adult life. We observed that the flies whose parents were exposed to levodopa at 6 h post-eclosion exhibited a similar overfeeding phenotype in adult life, independent of sex. However, overfeeding was differentially expressed for the offspring from flies treated at 3 days post-eclosion, as females, but not males, showed a significant increase in feeding. Interestingly, flies treated during at 5 days post-eclosion were unable to produce inheritable effects in feeding behavior. Moreover, for the flies that displayed overfeeding behavior, there was no clear pattern for the activity bout number and duration parameters for sex or age. These results indicate that transgenerational behavioral phenotypes may depend on the parental developmental stage when experiencing environmental stress, and can produce different effects for male and female offspring.

In humans, results from the Dutch Winter study indicate that famine exposure during development is more likely to induce a transgenerational increase in neonatal adiposity [12]. Additionally, data indicates a differential expression in the prevalence of over-eating disorders between sexes, as a recent survey with 9282 Americans show that 3.5% of women and 2.0% of men had binge eating disorder during their life [31]. The observed behavioral phenotypes from transient dopamine imbalance in flies are in line with these characteristics, suggesting that this model might be a reliable translational model for inheritable traits, especially when investigating the role of the dopaminergic system in eating disorders.

To date, most *Drosophila* models of transgenerational obesity-like characteristics focused on how parental diet misbalances can cause inheritable endocrine modifications, such as changes in lipid and carbohydrate metabolic pathways [32]. Promising molecular models have been proposed to explain these phenotypes, such as diet-induced variations in ribosomal DNA [33] or modifications in chromatin state [34], which correlate with offspring genome alterations. These adjustments include abnormal expression of several genes, some involved in energy metabolism, and many of unknown function [32]. Nevertheless, the current interpretation of these results is limited, as there is little elucidation of how physiological systems are affected by these molecular factors. For example, it is possible that epigenetic modifications necessary for the homeostatic regulation of synaptic receptors [35] could induce inheritable behavioral traits, a phenomenon already observed in rodent models of prenatal stress [36]. Our data indicates that the *Drosophila melanogaster* can be a useful tool for future research investigating these hypotheses.

To the best of our knowledge, our study is the first to demonstrate that the dopaminergic system may have a role in the development of obesity-like hereditary phenotypes, as it modulates the central regulation of food intake. Specifically, we show that the inheritability of this overfeeding behavior is differentially regulated depending on the parental age of treatment. As little is known about dopaminergic circuitry characteristics at different developmental stages in *Drosophila*, further research is needed to elucidate the factors that contribute to the transmission of this behavior across generations. Thus, these results can pave the way for future investigations of the interplay between environment-induced genetic changes and nervous system regulation of behavior when evaluating transgenerational traits.

## Figures and Tables

**Table 1:** Dopaminergic administration does not influence general locomotion. Levodopa treatment was delivered in the food for 2 days to 6 h, 3 days or 5 days post-eclosion (p.e.) flies before the behavioral testing at 7-days old, as described in Figure 1A. There were no observable differences between any group of levodopa-treated flies and the respective control, either for movements or sleep counts (Student *t* test; alpha = 0.05). Sample sizes are the same as shown in Figure 1.

		Movement Counts (Daily Average ± SEM)		Sleep Counts (Daily Average ± SEM)	
		Males	Females	Males	Females
6 h p.e.	Control	536.6 ± 45.88	457.9 ± 36.7	1053 ± 204.8	1083 ± 210.7
	Levodopa	597.6 ± 45.3	407.2 ± 33.2	1031 ± 155.3	1050 ± 193.4
	*p*-value	0.3490	0.3077	0.5850	0.4629
3 days p.e.	Control	654.8 ± 41.5	522.5 ± 50.4	1030 ± 169.3	1047 ± 208.9
	Levodopa	644.4 ± 49.0	542.5 ± 43.2	1030 ± 142.5	1031 ± 186.6
	*p*-value	0.8704	0.7635	0.9883	0.6927
5 days p.e.	Control	617.6 ± 50.1	493.1 ± 36.3	1037 ± 168.3	1017 ± 210.1
	Levodopa	565.6 ± 52.9	507.7 ± 66.3	1036 ± 182.9	1076 ± 184.8
	*p*-value	0.5187	0.8516	0.9719	0.1871

## Supplementary Materials

The following are available online at https://www.mdpi.com/2076-3425/10/8/487/s1, Table S1: Least-squares multiple linear regression model results. The table show estimates of the overall effect for the number of sips parameter, of treatment (0 = control; 1 = levodopa), sex (0 = male; 1 = female), and age (1, 3 or 5 days old) factors, for the F0 and F1 generations, Table S2: Levodopa increases the number of activity bouts, but differentially modulates its duration. Treatment was delivered in the food for 2 days to 6 h, 3 days or 5 days post-eclosion (p.e.) flies before the behavioral testing at 7-days old, as described in Figure 1A. All groups exhibited higher number of activity bouts, although the difference for flies treated 3 days p.e. did not reach statistical significance. The average activity bout duration was significantly higher for female groups only (Student *t* test; alpha = 0.05). Sample sizes are the same as shown in Figure 1, Table S3: Offspring of levodopa-treated flies at 6 h, 3 days or 5 days post-eclosion (p.e.) have altered feeding behavior depeding on sex and age of parental treatment. Female offspring of flies treated during early adulthood (6h and 3d p.e.) exhibited significantly higher number of activity bouts, while the activity bout duration was increased for 3d-p.e. F_1_ females and 6h-p.e. F_1_ males (Student *t* test; alpha = 0.05). Sample sizes are the same as shown in Figure 2.
