# Supplementary material for: Transient Administration of Dopaminergic Precursor Causes Inheritable Overfeeding Behavior in Young Drosophila melanogaster Adults
Source: Brain Sci. 2020 Jul 28;10(8):487. doi: 10.3390/brainsci10080487 (PMC7465534; doi:10.3390/brainsci10080487)
Supplement: Supplementary file 1 [file brainsci-10-00487-s001.zip › Supp Files/Supp Table 1.docx]

**Supp. Table 1.** Least-squares multiple linear regression model results. The table show estimates of the overall effect for the number of sips parameter, of treatment (0 = control; 1= levodopa), sex (0 = male; 1= female), and age (1, 3 or 5 days old) factors, for the F0 and F1 generations.

| **Number of sips regression for the F_0_ generation** | | | | |
| --- | --- | --- | --- | --- |
| **Variable** | **Estimate** | **SEM** | **95% CI** | **p- value** |
| Intercept | 56,96 | 55,62 | -71,30 to 185,2 | 0,3357 |
| Age | 17,36 | 13,49 | -13,75 to 48,47 | 0,2341 |
| Sex | -22,22 | 44,06 | -123,8 to 79,37 | 0,6276 |
| Treatment | 162,6 | 44,06 | 61,03 to 264,2 | 0,0061 |
| **Number of sips regression for the F_1_ generation** | | | | |
| **Variable** | **Estimate** | **SEM** | **95% CI** | **p- value** |
| Intercept | 295,2 | 75,05 | 122,2 to 468,3 | 0,0043 |
| Age | 24,04 | 18,20 | -17,94 to 66,01 | 0,2232 |
| Sex | 14,68 | 59,45 | -122,4 to 151,8 | 0,8111 |
| Treatment | 154,3 | 59,45 | 17,23 to 291,4 | 0,0318 |
