# Supplementary material for: Transient Administration of Dopaminergic Precursor Causes Inheritable Overfeeding Behavior in Young Drosophila melanogaster Adults
Source: Brain Sci. 2020 Jul 28;10(8):487. doi: 10.3390/brainsci10080487 (PMC7465534; doi:10.3390/brainsci10080487)
Supplement: Supplementary file 1 [file brainsci-10-00487-s001.zip › Supp Files/Supp Table 2.docx]

**Supp. Table 2.** Levodopa increases the number of activity bouts, but differentially modulates its duration. Treatment was delivered in the food for 2 days to 6 hours, 3 days or 5 days post-eclosion (p.e.) flies before the behavioral testing at 7-days old, as described in Fig 1A. All groups exhibited higher number of activity bouts, although the difference for flies treated 3 days p.e. did not reach statistical significance. The average activity bout duration was significantly higher for female groups only (Student t test; alpha = 0.05). Sample sizes are the same as shown in Fig 1.

|  |  | **Activity bout number**  **(Total occurrences ± SEM)** | | **Activity bout duration**  **(Average in seconds ± SEM)** | |
| --- | --- | --- | --- | --- | --- |
|  |  | Males | Females | Males | Females |
| 6 hours p.e. | Control | 20.0 ± 3.4 | 12.1 ± 3.2 | 4.7 ± 0.32 | 2.8 ± 0.33 |
|  | Levodopa | 35.3 ± 5.8 | 31.1 ± 6.5 | 4.5 ± 0.28 | 4.6 ± 0.39 |
|  | p-value | 0.0467 | 0.0132 | 0.6994 | 0.0019 |
| 3 days p.e. | Control | 14.6 ± 4.4 | 12.6 ± 3.5 | 3.8 ± 0.45 | 3.4 ± 0.41 |
|  | Levodopa | 32.2 ± 9.0 | 25.7 ± 5.8 | 3.4 ± 0.47 | 5.7 ± 0.69 |
|  | p-value | 0.0764 | 0.0664 | 0.5480 | 0.0081 |
| 5-days p.e. | Control | 27.1 ± 4.4 | 13.6 ± 2.7 | 4.3 ± 0.21 | 3.1 ± 0.25 |
|  | Levodopa | 52.4 ± 7.7 | 36.4 ± 10.3 | 4.9 ± 0.24 | 4.7 ± 0.45 |
|  | p-value | 0.0131 | 0.0150 | 0.1091 | 0.0012 |
