# Supplementary material for: Transient Administration of Dopaminergic Precursor Causes Inheritable Overfeeding Behavior in Young Drosophila melanogaster Adults
Source: Brain Sci. 2020 Jul 28;10(8):487. doi: 10.3390/brainsci10080487 (PMC7465534; doi:10.3390/brainsci10080487)
Supplement: Supplementary file 1 [file brainsci-10-00487-s001.zip › Supp Files/Supp Table 3.docx]

**Supp. Table 3.** Offspring of levodopa-treated flies at 6 hours, 3 days or 5 days post-eclosion (p.e.) have altered feeding behavior depeding on sex and age of parental treatment. Female offspring of flies treated during early adulthood (6h and 3d p.e.) exhibited significantly higher number of activity bouts, while the activity bout duration was increased for 3d-p.e. F_1_ females and 6h-p.e. F_1_ males (Student t test; alpha = 0.05). Sample sizes are the same as shown in Fig 2.

|  |  | **Activity bout number**  **(Total occurrences ± SEM)** | | **Activity bout duration**  **(Average in seconds ± SEM)** | |
| --- | --- | --- | --- | --- | --- |
|  |  | Males | Females | Males | Females |
| 6 hours p.e.  offspring | Control | 34.6 ± 4.4 | 22.3 ± 3.2 | 5.3 ± 0.25 | 4.8 ± 0.30 |
|  | Levodopa | 44.1 ± 4.9 | 43.1 ± 5.7 | 6.5 ± 0.41 | 5.2 ± 0.26 |
|  | p-value | 0.1560 | 0.0026 | 0.0099 | 0.4413 |
| 3 days p.e.  offspring | Control | 46.3 ± 8.0 | 27.3 ± 4.5 | 5.4 ± 0.40 | 5.1 ± 0.29 |
|  | Levodopa | 45.9 ± 6.9 | 44.8 ± 5.5 | 5.6 ± 0.32 | 6.9 ± 0.53 |
|  | p-value | 0.9659 | 0.0180 | 0.7076 | 0.0057 |
| 5-days p.e.  offspring | Control | 37.0 ± 4.8 | 43.2 ± 4.9 | 5.9 ± 0.62 | 5.7 ± 0.48 |
|  | Levodopa | 37.9 ± 6.3 | 47.9 ± 5.8 | 5.9 ± 0.41 | 5.7 ± 0.32 |
|  | p-value | 0.9098 | 0.5477 | 0.9936 | 0.9914 |
